# Distributed Flexible Sensors Based on Supercapacitor Gel Materials

**DOI:** 10.3390/gels11020139

**Published:** 2025-02-16

**Authors:** Chenghong Zhang

**Affiliations:** School of Electronics & Information Engineering, Guiyang University, Guiyang 550005, China; zhangchenghongcn@aliyun.com or zhangchenghong@gyu.cn

**Keywords:** supercapacitor, ion gel, flexible sensor, sensing layer, localization layer

## Abstract

Gel material sensors are lightweight, have fast response speeds and low driving voltages, and have recently become a popular research topic worldwide in the bionics field. A sensing unit is formed by pressing two kinds of gel materials together: a positioning layer gel based on acrylamide and lithium chloride and a sensing layer gel based on the ionic liquid BMIMBF_4_. Based on a stress–strain experiment of the sensing layer gel, a constitutive relationship model of its hyperelastic mechanical properties was established, and the elastic modulus and Poisson’s ratio of the sensing layer material were deduced. The capacitive response of the ion‒gel shunt capacitor to loading was observed to prove its ability to act as a pressure sensor. Although the gel thickness differs, the capacitance and load pressure exhibit a linear relationship. The capacitance was measured via cyclic voltammetry using the equivalent plate capacitor model for the positioning layer gel. The capacitance range of the gel sensor of a certain size was obtained via the cyclic voltammetry integral formula, which provided parameters for circuit design. A plate capacitor model of the sensing layer gel and an open four-impedance branch parallel model of the positioning layer gel were established. Two confirmatory experiments were designed for the models: first, the relationship between the sensing layer force and capacitance was measured, and the function curve relationship was established via a black box model; second, the theoretical and measured points of the positioning layer were compared, and the error was analyzed and corrected.

## 1. Introduction

A flexible haptic sensor system is based on flexible materials, is thin and transparent, has good tensile properties, corrosion resistance, etc., and has strong advantages in terms of performance and structure. Ensuring the flexibility of materials is a precondition for preparing tactile sensors. Two methods are typically used to determine the tensile properties of artificial skin and ensure its softness [1]. First, a thin conductive material is directly bonded to a flexible substrate. Second, a stretchable assembly is prepared by fusing a conductive material with a material substrate. For example, in the study by Someya et al. [2], a fluorine-containing copolymer and conductive substances were mixed to form an elastic conductor ink for printing. The prepared organic light-emitting diode active matrix had strong tensile properties, and through the ionic liquid preparation of slender nanotubes, the stretchability increased by up to twofold, and the conductivity increased to 100 S·cm^−1^. Rogers et al. [3] first proposed an inorganic semiconductor and its related electronic elements and tested the assembled circuit in a stretchable device. The performance of the inorganic metal materials in the base elastic surface paste was good. The authors first proposed an island–bridge design structure with a rigid module as an island, a rigid small material as a connecting bridge, and a deformable part for noncoplanar connections.

The piezoelectric effect, resistance effect, and shunt capacitance are commonly used in flexible contact-sensing materials [4,5]. Shunt capacitors have the advantages of low temperature sensitivity, high stability, high natural frequency, good dynamic performance, and low power consumption [6]. The pressure is measured by the change in the distance between the electrodes caused by the load on the capacitor. Bao used microstructured polydimethylsiloxane (PDMS) as the dielectric layer of shunt capacitors to improve the compressibility of the material [7]. A typical shunt capacitor structure comprises at least three layers: electrode‒dielectric‒electrode. A difficulty in pressure-sensing materials for shunt capacitors is the adhesion between the electrode and the dielectric. Stretching and deformation can cause sliding or separation between layers, resulting in noisy signals or even disabling the sensing. Gel materials are known for their excellent tunable adhesion and force-matching properties [8]. Masarapu studied the effect of pressure on the capacitance of ionic gels [9], and Kim prepared soft materials for touch localization based on ionic hydrogels [10], indicating the great potential of gels for flexible sensing and device packaging.

In this work, a pressure-sensing material was prepared based on previous research on ionic gel actuators. For sensing materials with different gel thicknesses, a linear relationship between pressure and capacitance is realized. To realize a material that can sense touch and pressure, a touch-positioning material was fabricated. This material was then used to construct a novel multilayer material on top of the pressure-sensing material, with a layer of VHB tape sandwiched between them for signal isolation. A simple distributed sensing system was constructed from this multilayer material. Studies have shown that this new material has touch and pressure-sensing capabilities, indicating its great potential as a material for the integration of smart actuator sensors for soft robotics.

## 2. Results and Discussion

### 2.1. Gel Positioning Layer Experiment

**Figure 1:**
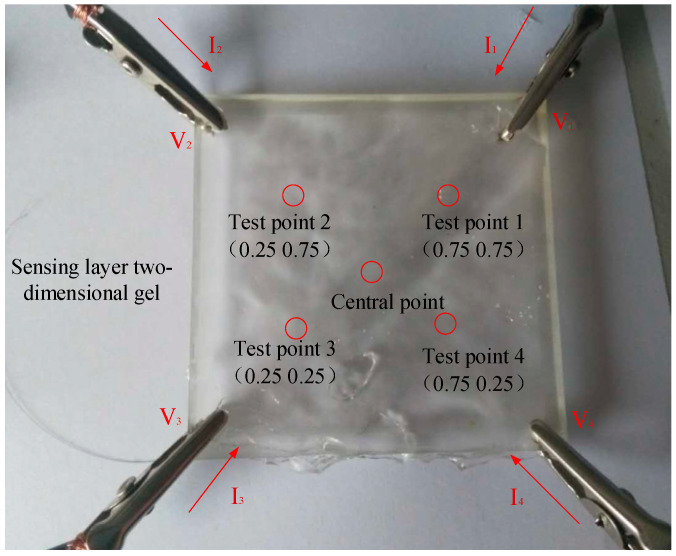
Points selected for the positioning layer test.

The center point of the colloid with respect to the four vertices is the reference point, and the center points along the diagonals are the test points, which are labeled test point 1 (0.75, 0.75), test point 2 (0.25, 0.75), test point 3 (0.25, 0.25), and test point 4 (0.75, 0.25). The coordinate (1, 1) corresponds to the first measurement branch, represented by V_1_; (0, 1) corresponds to the second measurement branch, represented by V_2_; (0, 0) corresponds to the third measurement branch, represented by V_3_; and (1, 0) corresponds to the fourth measurement branch, represented by V_4_. The test points selected for the positioning layer are shown in Figure 1.

**Figure 2:**
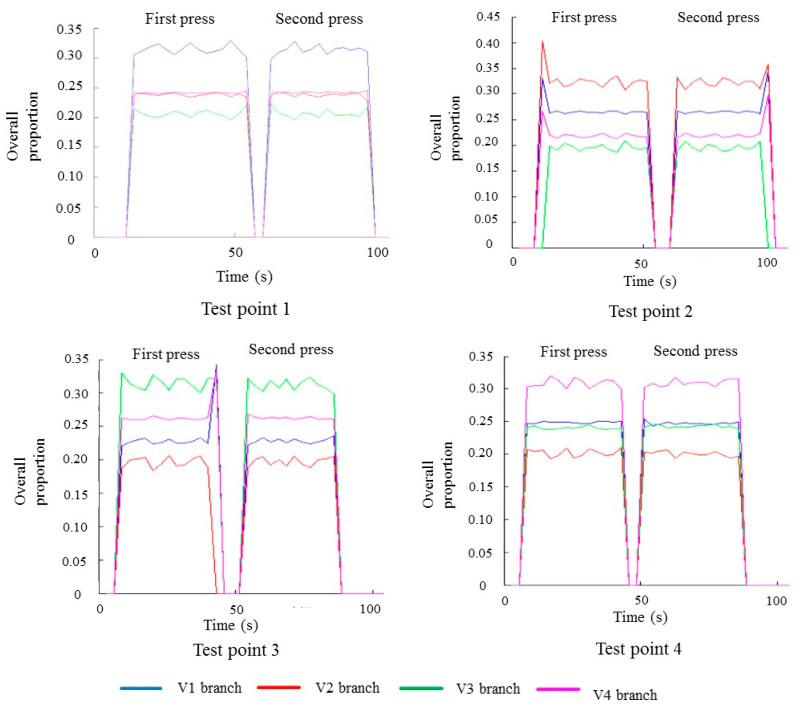
Effective data when the positioning layer is pressed.

Each test point was pressed twice for 1 min, and the interval was 15 s. The effective data when the positioning layer was pressed are shown in Figure 2.

The experiment shows that when there is no pressing, a parallel capacitor is formed between the colloid and the earth, which generates a small current signal, which can be considered noise. After pressing, the fingers touch the gel, the system forms a closed loop, and each branch circuit has a different pressing position and a different current. However, whether the positioning assumption of an equal ratio distribution is met cannot be intuitively observed, and data processing is needed.

### 2.2. Sensing Layer Gel Force–Capacitance Experiment

**Figure 3:**
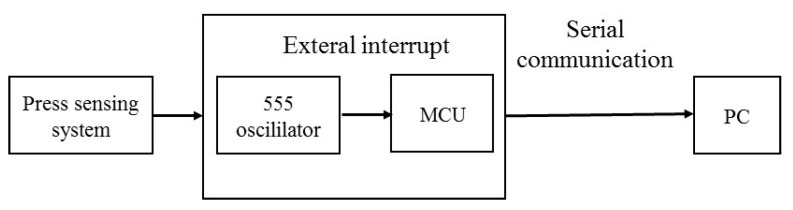
Perceptual layer measurement system.

The capacitance measured by the oscillation method is characterized by the use of the 555 chip to work directly in the unsteady region, and the frequency of the square wave output is a certain function of the measured capacitance in the circuit in series, so the capacitance value of the measured object can be calculated by measuring the frequency value of the output of the chip. Calculating the frequency value requires the use of the microcontroller’s counter and external interrupt function and then communication with the computer, as shown in Figure 3.

**Figure 4:**
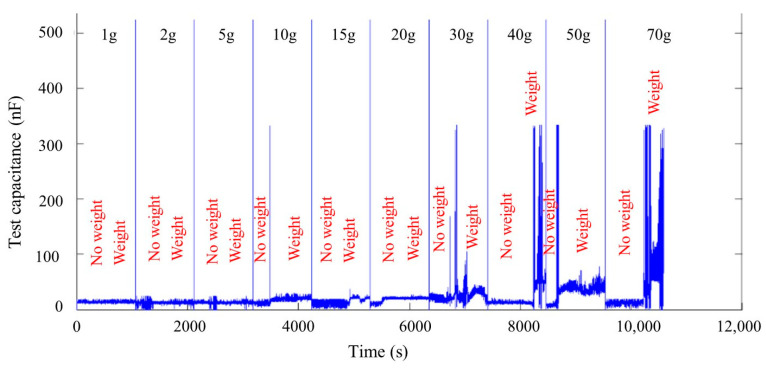
Experimental data of the force‒capacitance relationship of the sensing layer.

The size of the sensing layer was 45 mm × 16 mm × 0.8 mm, and weights of 1 g, 2 g, 5 g, 10 g, 15 g, 20 g, 30 g, 40 g, 50 g, and 70 g were placed on the ends as the experimental fixed force input. As the applied force increases, the capacitance of the sensing layer changes, and the relationship between the applied force and the capacitance is shown in Figure 4.

The capacitance under the stress state was filtered and averaged. The relationship between force and capacitance is shown in Table 1.

A characteristic of a sensor, e.g., the relationship between the input and output, is called a static characteristic when the input is constant or changes extremely slowly. A static property of the sensor means that the input quantity *x* does not change with time, and the functional relationship between the output quantity *y* and the input quantity *x* is

(1) $$y=a_{0}+a_{1}x+a_{2}x^{2}+a_{3}x^{3}+\ldots .a_{n}x^{n}$$

where *a* represents the calibration coefficient of the sensor, *x* represents the applied force, and *y* represents the capacitance change.

**Figure 5:**
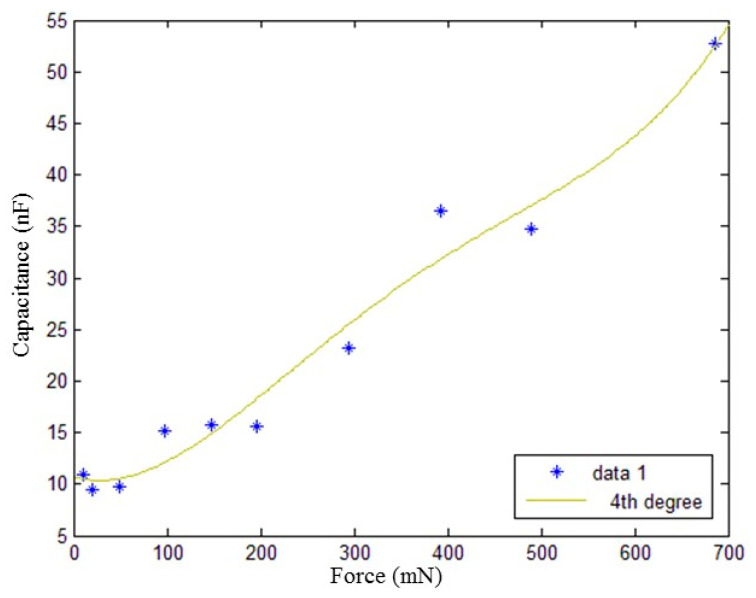
Capacitance–force curve of the sensing layer.

Ten pairs of data were measured through static experiments, and the fitting curve representing the static characteristic curve of the sensor was obtained. When fourth-order data fitting is used, the force and capacitance of the sensing layer basically meet the experimental requirements. The fitting curve of the perceived force and capacitance is shown in Figure 5.

The relationship model of the coupling between the capacitance and applied force of the sensing layer is as follows:

(2) $$y=8e^{-10}\times x^{4}-1.1e^{-6}\times x^{3}+0.00052\times x^{2}-0.027\times x+11$$

In the range of 0~686 mN, the capacitance and force can be expressed as a fourth-order polynomial.

Resolution refers to the minimum change in force that a sensor can detect, and when the change does not exceed this value, the sensor output will not change. In the case of a force of less than 10 mN, the sensor has essentially no response; with increasing applied force, the capacitance rapidly increases, the relationship between 200 mN and 700 mN is essentially linear, and the sensitivity is 6.396%/mN.

Based on multilayer ion gel materials, a micromodel of a distributed sensing system for soft robots is proposed. Distributed sensing emphasizes the measurement of the distribution of a certain physical stimulus between the subject and the environment through sensing units with multiple functions. The unit of the distributed sensing system we propose can sense contact and pressure without interference. Compared with a large area of a pressure-sensing material, a smaller area helps achieve uniform pressure on the sensing unit and improves the sensor performance. For the distributed sensing system studied in this paper, an intermediate-level sensing unit with position- and pressure-sensing capabilities is introduced. This kind of multilayer contact pressure-sensing ion gel material will promote the development of distributed sensing systems and control algorithms for soft robots and promote their research and application.

This type of sensor is thicker than some previously reported flexible sensors, such as [4,7,11,12], which is often undesirable. The thickness is attributed to a combination of aluminum foil, activated carbon, and a gel with a pressure-sensing layer, as well as a gel with a touch-positioning layer. The actuation capacity of this material is generated by a pressure-sensitive layer. To obtain integrated actuation capability for soft robots, a gel with a pressure-sensing layer needs to have a certain thickness to maintain its driving capacity, and an activated carbon layer is also needed. However, aluminum foil can be replaced with thicker conductors, such as printed silver nanowires (AgNWs) [13] and liquid metal alloy conductors (eutectic gallium indium, EGaIn), to reduce their thickness contribution. The gel thickness of the touch-positioning layer can also be reduced. In this work, the gel layer is achieved via soft molding. A thicker gel layer can be achieved by reducing the mold thickness.

## 3. Conclusions

Positioning layer gels based on acrylamide and lithium chloride and sensing layer gels based on the ionic liquid BMIMBF_4_ polymer are discussed in detail in this paper. Based on the equivalent plate‒capacitor relationship, the capacitance of the gel was measured via cyclic voltammetry, and the capacitance range of the gel was obtained via the cyclic voltammetry integral formula. The gel of the positioning layer can be equivalent to an open-circuit four-impedance branch parallel model. When the fingers touch the gel of the positioning layer, a complete loop is formed, and the contact position is judged based on the different currents of the four branches. The validity of the positioning layer design model was verified. A plate capacitor model was established for the sensing layer, and the capacitance changed with the distance between the plates. A functional relationship between the gel force and capacitance was established via experiments. Based on the stress–strain experiment of the sensing layer gel, a constitutive relationship model of the hyperelastic mechanical properties of the sensing layer was established, and the elastic modulus and Poisson’s ratio of the sensing layer were deduced from this model. The range of conditions for the use of the gel materials was determined to ensure the consistency of the properties of the gel materials.

In this work, we propose a multilayer contact-sensing ionization gel material suitable for the integrated drive of sensing soft robots. First, the pressure-sensing capability of the ionogel-based bilayer soft actuator was studied. Despite the difference in gel thickness, a linear relationship is achieved between capacitance and pressure. To locate the touch, a touch-positioning material was fabricated. On this basis, a new multilayer material was constructed on top of the pressure-sensing material, which was sandwiched in a layer of VHB tape for signal isolation. A study of a miniature distributed sensing system revealed that this material has good tactile sensing properties.

## 4. Materials and Methods

In this work, the flexible sensor includes a positioning layer and a sensing layer, which are combined as sensor components of artificial skin. The function of the positioning layer is to provide the position of the perceived force, and this layer enables the artificial skin to perceive the position relationship properties. It is mainly composed of polyacrylamide (PAAm) and lithium chloride (LiCl). The function of the sensing layer is to provide the magnitude of the perceived force, and this layer enables the artificial skin to perceive the force magnitude properties. It mainly includes a three-layer structure: the bottom ionic colloidal layer, which is mainly based on 1-butyl-3-methylimidazole tetrafluoroborate (BMIMBF_4_); the second layer of the active material, which comprises activated carbon powder (ACP) and activated carbon particles (ACGs); and the top fluid collection layer, which is made of aluminum foil with a thickness of 1 mm.

### 4.1. Ion Gel Sensor Unit Preparation

#### 4.1.1. Preparation of the Sensing Layer

**Figure 6:**
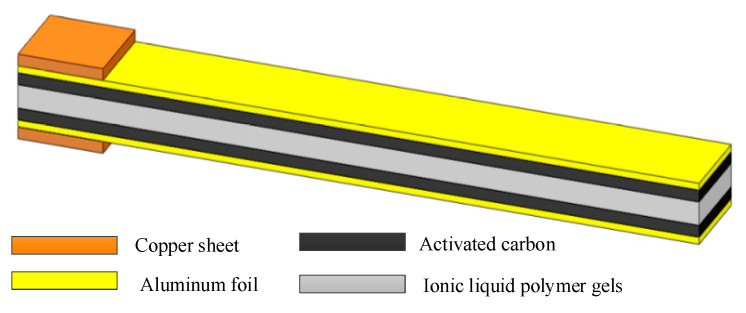
Ionic gel sensing layer model.

The sensing layer is divided into three parts: the ion gel layer is located in the middle, the activated carbon layer covers both sides of the ion gel, and the fluid-collecting aluminum foil covers the outermost layer. The ion gel mimics human skin and provides an ionic charge for the sensing gel. The activated carbon layer adsorbs the anions and ions in the ionic liquid, and the aluminum foil generates charge and has anti-folding and pressing functions. The ionic gel sensing layer model is shown in Figure 6.

The underlying ionic colloidal layer of choice is 1-butyl-3-methylimidazole tetrafluoroborate (BMIMBF_4_). The underlying ionogel carrier material is N,N-dimethylacrylamide (DMAA), which is chemically active and easy to copolymerize or homopolymerize with various monomers. Under the conditions of 2,2-diethoxyacetophenone (DEAP) and ultraviolet light, a mixed solution composed of BMIMBF_4_, DMAA, and 6,6′-diamino-3,3′-methyl dibenzoic acid (MBAA) underwent a polymerization reaction to form a pore-like solid structure, and the polymers were cross-linked with each other.

**Figure 7:**
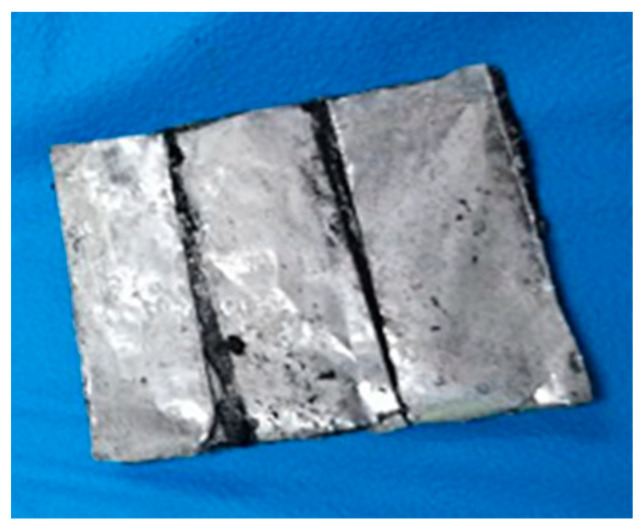
Ionic gel-sensing layer.

Activated carbon and carbon black are materials for the preparation of electrical grade. Activated carbon has a large specific surface area and strong adsorption, carbon black adsorbs gaseous hydrocarbons and other substances, and the mixture of the two adsorbs colloidal ions and can adsorb ion charges. Activated carbon and carbon black are formed by a 60% dispersion of polytetrafluoroethylene (PTFE) to form an adsorption carbon electrode. Eight grams of activated carbon and 1 g of carbon black were combined, stirred well, and mixed with 1 mL of PTFE to form a mixed solid mixture while 20 mL of water and 20 mL of ethanol were added. The mixture was stirred until the coagulation state was reached. The mixture was rolled repeatedly with a glass rod to form a pancake state with a thickness of approximately 0.5 mm, placed in a 120° oven, and shaken for 12 h to form the final drying electrode. The ionic gel-sensing layer is shown in Figure 7.

#### 4.1.2. Positioning Layer Preparation

The positioning layer is composed of a single layer of ionic gel, which is composed of a layer of conductive ionic gel, mainly acrylamide, and lithium chloride, and its advantage is that the positioning perception function can be completed under low-voltage conditions. Acrylamide is usually a colorless transparent sheet crystal with molecular weight and a schematic of its molecular structure; it is easily soluble in water and can be hydrolyzed into acrylic acid in an acid‒base environment. LiCl is a white cubic crystal or powder that provides conductive ions on the basal layer. Under the action of the crosslinker Bis, ammonium persulfate (AP) is added to aqueous solutions of acrylamide (Arc) and tetramethylethylenediamine (TEMED) to generate free radicals, and acrylamide and free radicals form activators, which polymerize with each other and finally form a long-chain structure.

**Figure 8:**
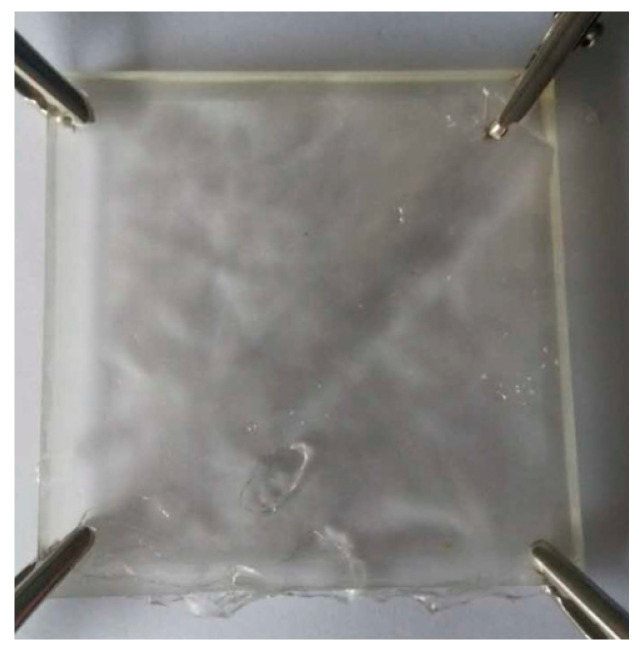
Ionic gel positioning layer.

A total of 14 wt% acrylamide monomer dissolved in water, the crosslinker N,N-methylenebis-acrylamide (MBAA), the heat initiator (potassium persulfate), and the catalyst TEMED were dissolved in an acrylamide solution at molar ratios of 0.028 mol%, 0.031 mol% and 0.152 mol%, respectively. To form a gel mixture, a 2 mol/L lithium chloride solution was used, and the dosage was adjusted according to the amount of gel. The solution was mixed into an acrylamide mixture, stirred evenly, and placed in a closed container with a length of 100 mm and a width of 100 mm. Four fixed points in the colloid were reserved for the colloidal positioning system, and the mixture was allowed to form a gel after standing at 50 °C for 3 h. The ionic gel positioning layer is shown in Figure 8.

### 4.2. Mechanical Properties of the Ion Gels

The initial and final states of a hyperelastic material determine its stored energy, which is unrelated to the material deformation process. This material is also known as a GREEN material. As a material with hyperelastic characteristics, the potential energy of acrylamide corresponding to the energy function can be expressed in terms of the stress *S* [14]:

(3) $$S=2\frac{\partial \psi (C)}{\partial C}=\frac{\partial U(E)}{\partial E}$$

where *Ψ* is the potential energy function.

The Green strain tensor *E* is defined as follows;

(4) $$ds^{2}-dS^{2}=2dX\cdot E\cdot dX$$

where *dS* is the length of the material before deformation and ds is the characteristic length after deformation. Adopting the component form of the tensor,

(5) $$dx_{i}dx_{i}-dX_{i}dX_{i}=2dX_{i}E_{ij}dX_{j}$$

To calculate the Green strain tensor, $dx_{i}dx_{i}$ can be written as follows:

(6) $$\begin{array}{l} dx\cdot dx=(F\cdot dX)\cdot (F\cdot dX)={\left(FdX\right)}^{T}(FdX)\\ =dX^{T}F^{T}FdX=dX\cdot (F^{T}F)\cdot dX \end{array}$$

where the part after the second equal sign is the matrix expression and *F* is the deformation gradient.

The differential is a small metric segment,

(7) dx=F⋅dX

In a two-dimensional coordinate system, the gradient is expressed as follows:

(8) $$E=\frac{1}{2}\left(F^{T}\cdot F-I\right)\mathrm{or}\;E_{ij}=\frac{1}{2}\left(F_{ik}^{T}\cdot F_{kj}-\delta_{ij}\right)$$

where *I* is the identity matrix and *δ_ij_* is the unit step function.

The energy function of hyperelastic materials can be independent of the deformation path, so there is a potential function. Taking the work conjugate of the second Pila–Kirchhoff stress tensor *S* with the Green strain *E*,

(9) $$\int_{E_{1}}^{E_{2}}SdE=U(E_{2})-U(E_{1})$$

Thus, the potential stress (potential energy) is path-independent and depends only on the starting state and the final state.

The potential strain energy (potential energy) of a superelastic material can be written as a function of the fundamental invariants (*I*_1_, *I*_2_, *I*_3_) of the deformation tensor, i.e., *Ψ* = *Ψ* (*I*_1_, *I*_2_, *I*_3_).

#### 4.2.1. Testing of the Electrochemical Properties of the Gels

The function of the sensing layer gel is to sense the capacitance change through the change in the charge of the ion gel that stores the charge. For a given sensitivity, detecting the charge change is easier when the electric charge storage capacity is high. The electric charge storage capacity of the sensing layer is a key parameter for measurement via the sensing layer gel.

The electric charge storage capacity of the sensing layer was measured via cyclic voltammetry. Triangular waves and voltages were loaded at different rates to enable the electrode–colloid redox reaction. The system automatically records the voltage and current in each state to determine whether the colloid undergoes redox reduction.

With respect to basic electrochemistry principles, an isosceles triangle-type pulse voltage [15] was loaded on the working electrode to obtain the voltage‒current relationship curve. If the scan starts from the cathode, then the first half indicates whether the electroactive substance undergoes a reduction reaction on the electrode, and the second half indicates whether the electrode undergoes an oxidation reaction. A complete cyclic voltammetry characteristic test involves a complete redox reaction process.

The average capacitance within any potential window is

(10) $$C=\frac{\int_{v_{1}}^{v_{2}}idV}{v(V_{2}-V_{1})}$$

The specific capacity is [13]

(11) $$C_{m}=\frac{C}{m}$$

where *m* is the mass (g) of the carbon electrode material.

The area is approximately equal to twice the above formula, where the capacitance is

(12) $$C_{sp}=\frac{2Q}{V\cdot u\cdot m}$$

where *C_sp_* represents the unit mass capacitance of the sensing layer, *Q* represents the integrated area of the cyclic voltammetry curve, *V* represents the voltage (V), *U* represents the scanning speed (V/s), and *m* represents the total mass (g) of the activated carbon.

The scanning speed determines the magnitude of the current; the two are proportional, and their ratio can be expressed by the interface double-layer capacitance parameter. In theory, the lower the scanning speed is, the closer the curve is to an ideal rectangle. Whether the assembled gel has a good ability to store charge can be determined. If the curves overlap or cross, then the charge storage capacity is zero, indicating that gel production has failed.

When the capacitance of the gel material is unchanged, the higher the scanning speed is, the higher the current. However, the transition time remains constant. When the specific capacity is taken as the ordinate unit, the faster the velocity is, the longer and narrower the curve is, and the more it deviates from the rectangular shape.

In this work, a CHI-660D potentiostat was selected for cyclic voltammetry measurements. In an environment of 25 °C, electrodes were loaded on both sides of the sensing layer with a size of 1 cm × 1 cm to test the electrochemical performance of the driver. The electrochemical window range is set to −3~3 V, and the gel can maintain stable chemical properties. Cyclic voltammetry curves were obtained at different scan speeds (5, 10, 25, and 50 mV/s).

#### 4.2.2. Nonlinear Superelastic Model of the Gel

For the colloid in the positioning layer, a polynomial constitutive relationship model is adopted, and the strain energy density can be expressed by formula (13):

(13) $$e=\sum_{i+j=1}^{N}C_{ij}{\left({\bar{I}}_{1}-3\right)}^{i}{\left({\bar{I}}_{2}-3\right)}^{j}+\sum_{i=1}^{N}\frac{1}{D_{i}}{\left(J-1\right)}^{2i}$$

where *I*_1_, *I*_2_, and *I*_3_ represent the strain invariants and where *C_ij_* represents the empirical parameters.

In the case of a small deformation of the gel, the expression is

(14) $$e=C_{10}({\bar{I}}_{1}-3)+C_{01}({\bar{I}}_{2}-3)+\frac{1}{D_{1}}{\left(J-1\right)}^{2}$$

The elastic constant is

(15) $$G=2(C_{10}+C_{01}),K=\frac{2}{D_{1}}$$

(16) $$E=\frac{9KG}{3K+G},V=\frac{3K-2G}{6K+2G}$$

where *E* represents the elastic modulus, *v* represents Poisson’s ratio, *G* represents the tangential force exchange, and K represents the elastic constant.

### 4.3. Gel Positioning Layer System Model

In the one-dimensional structure, polyacrylamide containing lithium chloride is used as an ion conductor, and platinum electrodes and a branch series ammeter are connected on both sides. The potentials on the two sides of the ion gel conductor are equal. When a finger touches a spot, because the body is connected with the earth, two parallel closed loops are formed at the conductor contact point, and the position of the contact point is expressed by the normalized distance equation.

The impedance (*Z*) values for the two paths in the colloid are

(17) $$Z_{1}=R_{1}-j\frac{1}{2\pi fC_{EDL}}$$

(18) $$Z_{2}=R_{2}-j\frac{1}{2\pi fC_{EDL}}$$

The resistance of the gel at a 0.1 mm thickness is approximately 10 kΩ, the capacitance of a finger is approximately 100 pF, and the *RC* delay of the strip is 10^−6^ s. When a touch screen is in a stable and noncontact state, theoretically, no current is present because there is no potential gradient on the panel.

The capacitance per unit area of the double layer is approximately 10^−1^ F/m^2^, the area is approximately 10^−5^ m^2^, and the power supply test frequency is 17 kHz:

(19) $$-j\frac{1}{2\pi fC_{EDL}}\approx -9j$$

Since the reactance is much smaller than the resistance of the ionic contact strip, the ratio between the two resistors is used to calculate the approximate contact current. When the fingers touch, the total current is

(20) $$I_{total}=\frac{V}{\frac{R_{1}R_{2}}{R_{1}+R_{2}}-j\frac{1}{2\pi fC_{finger}}}$$

(21) $$I_{1}\approx \frac{R_{2}}{R_{1}+R_{2}}I_{total}=(1-\alpha )I_{total}$$

(22) $$I_{2}\approx \frac{R_{1}}{R_{1}+R_{2}}I_{total}=\alpha I_{total}$$

Using the normalization method,

(23) $$(1-\alpha )=\frac{I_{1}}{I_{total}},\;\alpha =\frac{I_{2}}{I_{total}}$$

where *R* represents the total resistance, *α* is the normalized position, and each resistor is connected in series with the double-layer capacitor.

**Figure 9:**
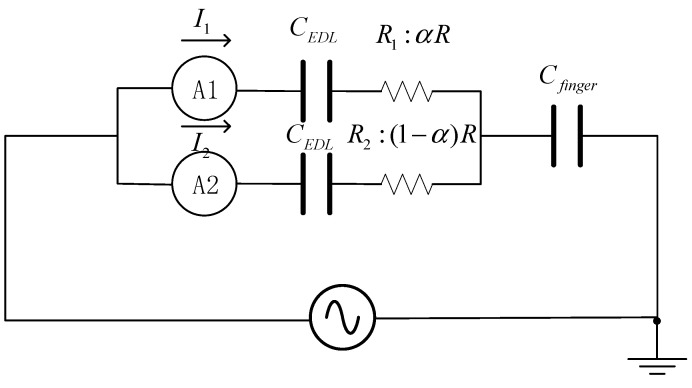
Equivalent circuit of the adhesive strip of the one-dimensional positioning layer.

The current through branches *I*_2_ and *I*_1_ is measured, the total current is *I* = *I*_1_ + *I*_2_, and the equivalent circuit of the one-dimensional positioning layer adhesive strip is shown in Figure 9.

**Figure 10:**
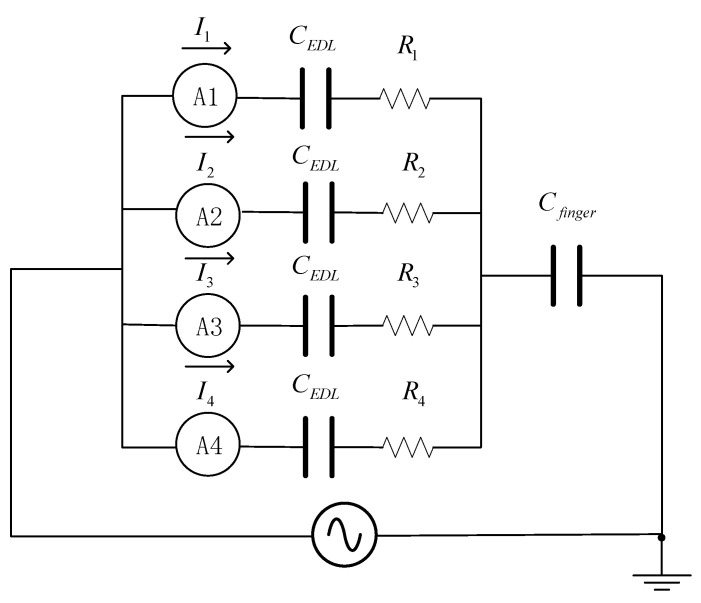
Equivalent circuit of the gel layer of the two-dimensional positioning layer.

When a finger touches the gel strip, the current contained in the electrode is transmitted to the outside through the finger, and the touch current delay is less than 20 ms. The contact current is proportional to the distance from the contact point to the electrode. When the *I*_1_ branch current decreases, the *I*_2_ branch current increases, and the total current remains unchanged. When the gel strip is stretched to twice its original size, the parasitic capacitance increases, the base current and the contact current also increase, and the current behavior is similar to negative linearity. The equivalent circuit of the gel layer of the two-dimensional positioning layer is shown in Figure 10.

When a finger touches the sensing layer, the finger contact is divided into 4 parts. In the equivalent circuit, the four virtual branches are in parallel, and the current of each branch is proportional to the distance between the contact point and the electrode. The position of the contact point can be calculated via the currents.

A contact point test was performed on a two-dimensional gel, and there were four contact points in total, as shown in Figure 3. (*α*, *β*) represent the horizontal and vertical coordinates of the measuring points; the coordinates in the lower left corner are (*α*_0_, *β*_0_) = (0,0), and the coordinates in the upper right corner are (*α*_4_, *β*_4_) = (1,1). The coordinates of the contact point are

(24) $$\alpha =\frac{I_{1}+I_{4}}{I_{1}+I_{2}+I_{3}+I_{4}}$$

(25) $$\beta =\frac{I_{1}+I_{2}}{I_{1}+I_{2}+I_{3}+I_{4}}$$

### 4.4. Sensing Layer Gel Model

**Figure 11:**
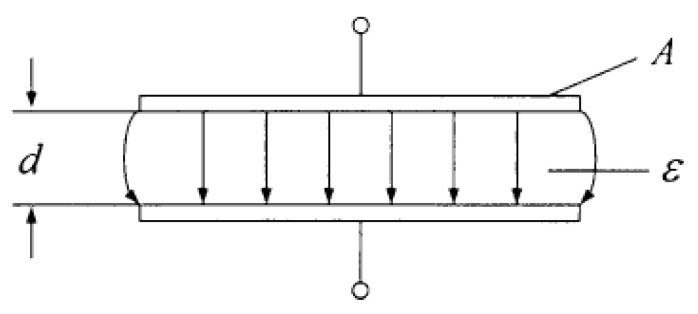
Plate capacitor model of the sensing layer gel sensor.

The sensing layer gel can be simplified as a force sensor model, and the working principle of the sensing layer is described with flat plate capacitors, as shown in Figure 11.

When the edge effect is ignored in the capacitance model,

$$Q=0.0279,v=6V,U=0.0025V/s,m=0.83g,C_{sp}=8.9\times 10^{-8}F$$

where ε represents the permittivity between capacitors, ε*_0_* represents the vacuum permittivity, and ε*_r_* represents the relative permittivity (value of 8.85 × 10^−12^ F/m).

Suppose that the initial capacitance of the sensor is *C*_0_; if the plate spacing of sensor *d* decreases from the initial value by ∆*d*_0_, then the capacitance *C* is

(26) $$C=C_{0}+\Delta C=\frac{\varepsilon_{r}\varepsilon_{0}A}{d_{0}-\Delta d}=\frac{C_{0}}{1-\frac{\Delta d}{d_{0}}}=\frac{C_{0}(1+\frac{\Delta d}{d_{0}})}{1-{\left(\frac{\Delta d}{d_{0}}\right)}^{2}}$$

The capacitance and the change in the plate spacing are linearly related, and the relative change in the capacitance is

(27) $$\frac{\Delta C}{C_{0}}=\frac{\frac{\Delta d}{d_{0}}}{1-\frac{\Delta d}{d_{0}}}$$

According to the Taylor series expansion,

(28) $$\frac{\Delta C}{C_{0}}=\frac{\Delta d}{d_{0}}[1+\frac{\Delta d}{d_{0}}+{\left(\frac{\Delta d}{d_{0}}\right)}^{2}+{\left(\frac{\Delta d}{d_{0}}\right)}^{3}+\ldots ]$$

When $\left|\frac{\Delta d}{d_{0}}\right|<<1$, the following linear relationship is obtained:

(29) $$\frac{\Delta C}{C_{0}}\approx \frac{\Delta d}{d_{0}}$$

The sensitivity of the capacitive sensor is

(30) $$S=\frac{\Delta C/C_{0}}{\Delta F}\approx \frac{1}{d_{0}}.\frac{\Delta d}{\Delta F}$$

The advantages of capacitive sensors are as follows:

(1) Their response speed is fast, their natural frequency of sensors of the capacitor structure class is very high, and they can work at a vibration frequency as low as a few hertz.
(2) Their precision is high, and their input energy level is very low, which is suitable for detecting slight force signals; displacements of 10 nm or less can be measured.
(3) Their structure is simple, and high accuracy can be achieved in various situations.
(4) These sensors are less affected by temperature, and the capacitance depends on the size of the sensor and is unrelated to the electrode material. Their heating is low, and their performance is stable.

**Figure 12:**
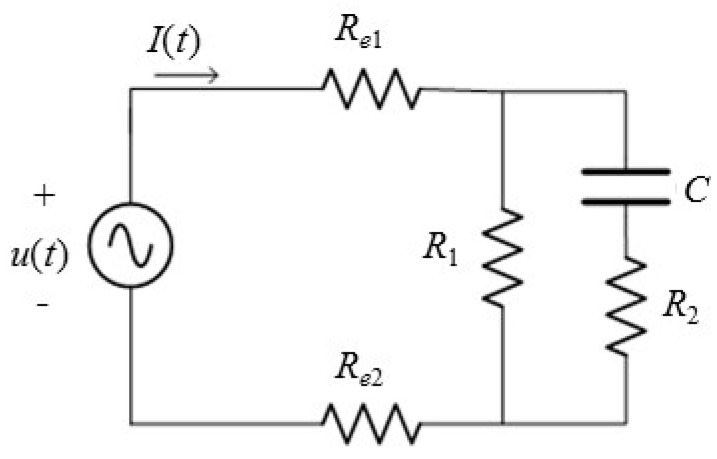
Equivalent circuit for the plate capacitor model of the sensing layer gel.

The conduction process of an ionic liquid polymer gel actuator can be summarized as the comprehensive effect of the ionic current, displacement current, and electronic current. From the perspective of describing the steady-state/dynamic characteristics of the actuator, the flexible ionic liquid polymer gel actuator can be simplified into an *RC* equivalent circuit model. The equivalent circuit of the ion gel in the sensing layer is shown in Figure 12:

where *R*_e1_ and *R*_e2_ represent the resistance of the upper and lower bipolar plates, respectively, *R*_1_ represents the total DC equivalent resistance of the activated carbon layer of the actuator and the ionic liquid polymer gel layer, *R*_2_ represents the dynamic equivalent resistance of the actuator, and *C* represents the actuator capacitance.

When the oscillation method is used to measure the capacitance, the frequency of the square wave output and the capacitance measured in series in the circuit have a functional relationship, and the capacitance of the measured object can be calculated by measuring the output frequency.

The charging and discharging times of the capacitor measured via the oscillation method are as follows:

(31) $$t_{pl}=R_{2}C_{x}In2$$

(32) $$t_{ph}=(R_{1}+R_{2})C_{x}In2$$

The square wave frequency is

(33) $$f=1/(t_{pl}+t_{ph})$$

The capacitance is

(34) $$C_{x}=1.44/(R_{1}+2R_{2})f$$

## Figures and Tables

**Table 1:** Relationship between the force and capacitance of the sensing layer.

Weight Mass (g)	Capacitance (nF)	Weight Mass (g)	Capacitance (nF)
1	10.89	20	15.54
2	9.43	30	23.13
5	9.68	40	36.48
10	15.1	50	34.72
15	15.8	70	52.71

## Data Availability

The original contributions presented in this study are included in the article. Further inquiries can be directed to the corresponding author.
